# FCGR2A and FCGR3A Genotypes in Human Immunodeficiency Virus Mother-to-Child Transmission

**DOI:** 10.1093/ofid/ofv149

**Published:** 2015-10-14

**Authors:** Caitlin Milligan, Barbra A. Richardson, Grace John-Stewart, Ruth Nduati, Julie Overbaugh

**Affiliations:** 1 Division of Human Biology; 2 Vaccine and Infectious Disease Division, Fred Hutchinson Cancer Research Center; 3 Medical Scientist Training Program, University of Washington School of Medicine; 4 Departments of Global Health; 5 Biostatistics; 6 Medicine; 7 Epidemiology; 8 Pediatrics, University of Washington, Seattle; 9 Department of Pediatrics and Child Health, University of Nairobi, Kenya

**Keywords:** Fc gamma receptors (FcγR), FCGR2A, FCGR3A, HIV, mother-to-child transmission (MTCT)

## Abstract

***Background.*** Fc-mediated effector functions have been suggested to influence human immunodeficiency virus (HIV) acquisition and disease progression. Analyzing the role of host Fc gamma receptor (FcγR) polymorphisms on HIV outcome in mother-to-child transmission (MTCT) will increase our understanding of how host genetics may alter immune responses in prevention, therapy, and disease. This study analyzed the impact of FCGR2A and FCGR3A genotypes on MTCT in a cohort in which Fc-mediated antibody functions are predictive of infant HIV outcome.

***Methods.*** Human immunodeficiency virus-positive mothers and their infants from a historical MTCT cohort were genotyped for FCGR2A and FCGR3A. We assessed the impact of these genotypes on transmission and acquisition of HIV and disease progression using χ^2^ tests, survival analyses, and logistic regression.

***Results.*** Among 379 mother-infant pairs, infant FCGR2A and FCGR3A genotypes were not associated with infant HIV infection or disease progression. Maternal FCGR2A was not associated with transmission, but there was a trend between maternal FCGR3A genotype and transmission (*P* = .07). When dichotomizing mothers into FCGR3A homozygotes and heterozygotes, heterozygotes had a 64.5% higher risk of transmission compared with homozygotes (*P* = .02). This risk was most evident in the early breastfeeding window, but a trend was only observed when restricting analyses to breastfeeding mothers (hazards ratio, 1.64; *P* = .064).

***Conclusions.*** Infant FCGR2A and FCGR3A genotypes were not associated with HIV infection or disease progression, and, thus, host FcγR genotype may not significantly impact vaccination or therapeutic regimens that depend on Fc-mediated antibody functions. Maternal FCGR3A genotype may influence early breastfeeding transmission risk, but more studies should be conducted to clarify this association and its mechanism.

## Abstract (precis)

Host FcγR polymorphisms may influence HIV infection and disease progression. Here, we examine FCGR2A and FCGR3A genotypes in HIV mother-to-child transmission. Infant genotypes did not impact infection or progression, but the maternal FCGR3A genotype may influence early breastfeeding transmission risk.

Recent data from human and macaque studies suggest that Fc-mediated antibody functions, such as antibody-dependent cellular cytotoxicity (ADCC), may protect from human immunodeficiency virus (HIV) infection and/or disease progression (reviewed in [1]). These functions depend on host Fc gamma receptors (FcγRs) that bind the Fc portion of antibodies, and single-nucleotide polymorphisms (SNPs) in these receptors can affect immunoglobulin (Ig)G binding affinity. Accordingly, these SNPs can alter the ability of effector cells bearing FcγRs to facilitate Fc-mediated antibody functions [2–7].

Two polymorphic FcγRs of interest are FcγRIIa and FcγRIIIa. FcγRIIa is an activating receptor found on many cell types: monocytes, macrophages, neutrophils, and platelets, among others. The receptor has a polymorphism (SNP rs1801274) in its gene (FCGR2A) that encodes either a histidine (H) or arginine (R) at amino acid position 131 [8, 9]. The H allele is associated with higher affinity IgG binding [5, 7, 9]. FcγRIIIa is less widely expressed than FcγRIIa, but it is the major FcγR found on natural killer cells, a key mediator of ADCC. The FcγRIIIa gene (FCGR3A) similarly encodes a SNP that alters binding affinity (SNP rs396991). FCGR3A encodes a valine (V) or phenylalanine (F) at codon 158, and the V form has higher affinity for IgG [5, 10, 11]. Cells expressing high-affinity receptors mediate higher levels of Fc-mediated effector functions compared with cells expressing their lower affinity counterparts [2, 4, 6]. However, studies examining the impact of these FCGR2A and FCGR3A polymorphisms on HIV acquisition and progression have variable results [2, 3, 12–20].

These FcγR genotypes may be particularly relevant to mother-to-child transmission (MTCT) because ADCC has been implicated in transmission risk and in infected infant disease course [21, 22]. This study addresses the impact of FCGR2A and FCGR3A polymorphisms on MTCT in a historical cohort in which ADCC has been described as a correlate of protection [21, 22]. In this study, we show that infant genotypes are not associated with HIV infection or progression. In mothers, FCGR2A genotype did not influence transmission risk, but there was evidence of FCGR3A genotype impacting early postpartum MTCT.

## METHODS

### Study Design

Antiretroviral naive mother-infant pairs from the Nairobi Breastfeeding Trial [23] were selected for genotyping based on availability of HIV infection outcome and a sample for genotyping (N = 379). At the time of the original trial, antiretrovirals were not the standard of care in Kenya, and thus no mothers or infants received treatment for prevention of MTCT. Infants were tested for HIV DNA at birth, 6 weeks, 14 weeks, 6 months, and every 3 months thereafter until 2 years of age. For those infants who tested positive, samples prior to the first HIV DNA positive test were screened for HIV RNA to more precisely define infection timing. Time of infant infection was estimated as the midpoint between the last negative HIV DNA or RNA test and the first positive test. Regular sampling allowed for accurate estimation of infection timing and progression in infants who became infected. Additional data on pregnancy and delivery were available for the cohort [23–25]. Maternal RNA plasma viral loads from enrollment during pregnancy (N = 362) were used for calculations [23, 24]. If maternal viral load from pregnancy was not available (N = 17), the first available viral load after delivery was used. Breast milk viral loads were available for 265 women [25]. The earliest breast milk viral load available was used for each woman; the majority of samples were from the first 6 weeks after delivery (N = 244), and the remaining samples were from week 7 to month 7 after delivery (N = 21). For viral loads less than the assay cutoff, the viral load was set as the midpoint between 0 and the cutoff. The ethical review committee of the Kenyatta National Hospital Institutional Review Board, the Institutional Review Board of University of Washington, and the Institutional Review Board of the Fred Hutchinson Cancer Research Center approved the study.

### FCGR2A and FCGR3A Genotyping

DNA extracted from blood (peripheral blood mononuclear cells or filter paper), breast milk, or cervical and vaginal samples was available for most individuals [26–29]. If not, DNA was extracted from plasma (31 mothers, 9 infants) using QIAGEN DNEasy or filter paper (6 infants) using the QIAGEN QIAamp DNA Mini Kit, per the manufacturer's protocol.

FCGR2A and FCGR3A genotypes were determined using TaqMan SNP Genotyping Assays C_9077561_20 and C_25815666_10, respectively (Life Technologies). TaqMan assays were previously verified in the laboratory by sequencing a subset of samples [18]. Control DNAs of known genotypes (determined by Sanger sequencing) were included in each TaqMan assay (Coriell Cell Repository).

### Statistical Analysis

χ^2^ tests for categorical variables and *t* tests with Welch's correction for comparisons of means were used to determine which cohort characteristics were associated with HIV infection and transmission. The associations between FCGR2A and FCGR3A genotypes and infection and transmission were first analyzed using χ^2^ tests for independence. Logistic regressions controlling for appropriate covariates (maternal plasma viral load, breastfeeding status, and infant prematurity) were used to determine associations between FcγR genotype and infection risk. The associations between viral load and genotype were analyzed by linear regression. Cox proportional hazards models and Kaplan-Meier estimates with log-ranks tests were used to determine the association between genotype and time to infection/time to infant mortality. A Pearson's χ^2^ test was used to determine whether SNPs were in Hardy-Weinberg equilibrium and to determine linkage disequilibrium between the two SNPs. Viral loads were log_10_ transformed for all analyses. Analyses were not adjusted for multiple comparisons because our main findings (on the impact of genotypes on infection/transmission and infant progression), set a priori, were not statistically significant [30]. We then performed additional exploratory analyses to further explore a potential mechanism behind the statistical trend observed with the association between maternal FCGR3A genotype and transmission.

## RESULTS

### Study Population Characteristics

In this study, 379 mothers and their corresponding infants from the Nairobi Breastfeeding Trial [23] were genotyped for FCGR2A and FCGR3A. Overall, there were 87 infant infections. Mothers who transmitted the virus to their infants had higher plasma viral loads (4.96 vs 4.47 log_10_ copies/mL, *P* < .0001), lower CD4 counts (360 cells/mm^3^ vs 447 cells/mm^3^, *P* = .0002), and were more likely to be in the breastfeeding arm of the original study (64.4% vs 45.2%, *P* = .002) (Table 1). In this cohort (which included in utero, delivery, and breastfeeding transmissions), maternal age, gravidity, delivery type (vaginal vs Cesarean section), prolonged membrane rupture (≥4 hours), and labor duration were not significantly associated with transmission risk. Human immunodeficiency virus-infected infants were more likely to be premature (12.7% vs 4.6%, *P* = .029), and there were more deaths during follow-up in infected infants than uninfected infants (44.8% vs 10.3%, *P* < .0001). Infected infants had an average set point viral load of 5.85 log_10_ copies/mL. These characteristics are similar to those found in the larger trial cohort [23, 24].

**Table 1.** Infant and Maternal Cohort Characteristics^a^

Variable	HIV-Infected Infants	HIV-Uninfected Infants	*P* Value
Number	87	292	
Premature (<37 wks)	7 of 55 (12.7%)	9 of 196 (4.6%)	.029
Low birth weight (<2500 g)	8 of 83 (9.64%)	16 of 267 (5.99%)	.25
Death during 2-yr follow-up	39 of 87 (44.8%)	30 of 292 (10.3%)	<.0001
Mean plasma set point viral load (log_10_ copies/mL)	5.85 (0.86)	NA	
	HIV-Transmitting Mothers	HIV-Nontransmitting Mothers	
Number	87	292	
Mean age	23.66 (4.09)	23.96 (4.37)	.56
Mean plasma RNA viral load (log_10_ copies/mL)	4.96 (0.65)	4.47 (0.83)	<.0001
Mean CD4 count (cells/mm^3^)	360 (171)	447 (224)	.0002
Mean gravidity	2.48 (1.61)	2.31 (1.39)	.37
Vaginal delivery	77 of 87 (88.5%)	264 of 286 (92.3%)	.27
Prolonged membrane rupture (≥4 h)	35 of 86 (40.7%)	92 of 279 (33.0%)	.19
Mean labor duration	10.18 (5.86)	10.95 (7.38)	.32
Breastfeeding arm of original trial	56 of 87 (64.4%)	132 of 292 (45.2%)	.002
Mean breast milk RNA viral load (log_10_ copies/mL)	3.13 (0.90)	2.76 (0.81)	.004

Abbreviations: HIV, human immunodeficiency virus; NA, not applicable.

^a^ Data are represented as number (percentage) or mean (standard deviation). *P* values are from χ^2^ tests of categorical variables and *t* tests with Welch's correction for comparisons of means.

### FCGR2A and FCGR3A Genotype Distributions

Of the 379 infants genotyped for FCGR2A, 88 (23.2%) were homozygous for the high-affinity allele (H/H), 178 (47.0%) were heterozygous (H/R), and 113 (29.8%) were homozygous for the low-affinity allele (R/R). Mothers had similar distributions of FCGR2A alleles: 88 (23.2%) H/H, 174 (45.9%) H/R, and 117 (30.9%) R/R. For the FCGR3A genotype, 41 (10.8%) infants were homozygous for the high-affinity allele (V/V), 173 (45.6%) were heterozygous (V/F), and 165 (43.5%) were homozygous for the low-affinity allele (F/F). Mothers also had similar distributions of FCGR3A alleles: 44 (11.6%) V/V, 152 (40.1%) V/F, and 183 (48.3%) F/F. The sample population was in Hardy-Weinberg equilibrium for both FCGR2A (χ^2^ = 3.35, *P* = .07) and FCGR3A (χ^2^ = 0.48, *P* = .49), and there was some evidence of linkage disequilibrium for the 2 SNPs (χ^2^ = 11.36, *P* = .02), as has been reported by others [18, 31]. These FCGR2A and FCGR3A genotype distributions are similar to what has been reported in other populations, including those in Kenya [3, 12, 14, 18, 19].

### FCGR2A and FCGR3A Genotypes and Human Immunodeficiency Virus Risk

In a χ^2^ test, infant FCGR2A genotype was not associated with HIV infection status (*P* = .54; Table 2). Similarly, maternal FCGR2A genotype was not associated with transmission (*P* = .64). Maternal-infant FCGR2A genotype concordance was associated with reduced odds of infant infection (odds ratio [OR] = 0.59; 95% confidence interval [CI], .37–.96; *P* = .04); however, this relationship did not remain significant after adjusting for factors associated with infant infection (maternal plasma viral load, breastfeeding, infant prematurity) (OR = 0.60; 95% CI, .32–1.13; *P* = .11) (Table 3).

**Table 2.** Infant and Maternal Genotypes by Infection or Transmission Status^a^

Genotype	HIV-Infected Infant/ Transmitting Mother (Total N = 87)	HIV-Uninfected Infant/Nontransmitting Mother (Total N = 292)	χ^2^	*P* Value
Infant FCGR2A Genotype				
H/H	24 (27.6%)	64 (21.9%)	1.23	.54
H/R	38 (43.7%)	140 (48.0%)		
R/R	25 (28.7%)	88 (30.1%)		
Maternal FCGR2A Genotype				
H/H	23 (26.4%)	65 (22.3%)	0.90	.64
H/R	40 (46.0%)	134 (45.9%)		
R/R	24 (27.6%)	93 (31.8%)		
Infant FCGR3A Genotype				
V/V	9 (10.3%)	32 (11.0%)	0.66	.72
V/F	43 (49.4%)	130 (44.5%)		
F/F	35 (40.2%)	130 (44.5%)		
Maternal FCGR3A Genotype				
V/V	7 (8.0%)	37 (12.7%)	5.44	.07
V/F	44 (50.6%)	108 (37.0%)		
F/F	36 (41.4%)	147 (50.3%)		

Abbreviations: HIV, human immunodeficiency virus.

^a^Data represent number (percentage) of infected/uninfected infants or transmitting/nontransmitting mothers with indicated genotype.

**Table 3.** Association Between Infant/Maternal Genotype Concordance and Infant Infection Status^a^

			Univariate Analysis		Multivariate Analysis	
Genotype	Number of Infected Infants	Number of Uninfected Infants	OR (95% CI)	*P* Value	OR (95% CI)	*P* Value
FCGR2A						
Concordant	37	162	.59 (.37, .96)	.04	.60 (.32, 1.13)	.11
Nonconcordant	50	130	1 (Ref)			
FCGR3A						
Concordant	48	172	.86 (.53, 1.39)	.54	.84 (.45, 1.57)	.58
Nonconcordant	39	120	1 (Ref)			

Abbreviations: CI, confidence interval; MTCT, mother-to-child transmission; OR, odds ratio.

^a^“Concordant” represents mother/infant pairs with the same FCGR2A or FCGR3A genotype. “Nonconcordant” represents mother/infant pairs in which the mother and infant had different genotypes. The multivariate analyses were adjusted for factors associated with MTCT in the cohort: maternal plasma viral load, breastfeeding, and infant prematurity.

With regard to FCGR3A, infant genotype was not associated with HIV infection (*P* = .72; Table 2). Maternal-infant FCGR3A genotype concordance was not associated with transmission or infection (Table 3). However, there was a trend for an association between maternal FCGR3A genotype and transmission (*P* = .07; Table 2). We unexpectedly found that heterozygote mothers seemed to be at greatest risk of transmission. When dichotomizing mothers into FCGR3A heterozygotes (V/F) and homozygotes (V/V or F/F), in a Cox proportional hazards model, heterozygotes had a 64.5% increased risk of transmission compared with homozygotes (*P* = .02). Furthermore, in a logistic regression controlling for factors associated with maternal transmission (plasma viral load, breastfeeding, and infant prematurity), the heterozygote genotype (V/F) was significantly associated with increased odds of infant infection compared with FCGR3A low-affinity homozygotes (F/F) (OR = 2.17; 95% CI, 1.11–4.24; *P* = .02). However, when comparing mothers with at least 1 high-affinity allele (V/F or V/V) to those mothers with only the low-affinity allele (F/F), there was not a statistically significant association between genotype and transmission (*P* = .14), suggesting that the presence of the high-affinity allele alone was not associated with increased transmission. Infant FCGR3A heterozygotes were not at an increased infection risk compared with homozygotes in a Cox proportional hazards model (hazards ratio [HR], 1.18; *P* = .44).

**Figure 1.**
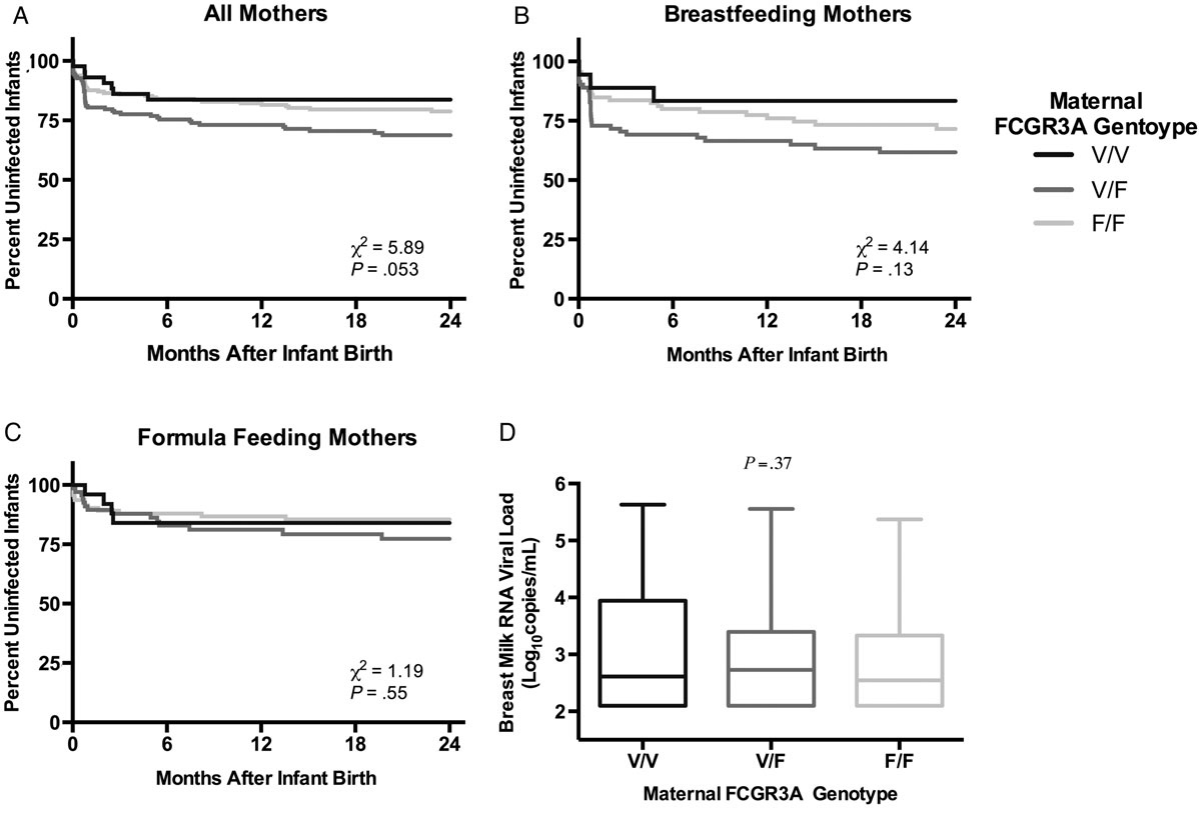
The impact of maternal FCGR3A genotype on time to transmission and breast milk viral loads. Kaplan-Meier estimates with log-rank statistics for time to infant infection by maternal FCGR3A genotype for all mothers (A), breastfeeding mothers (B), and formula feeding mothers (C). Breast milk RNA viral loads by maternal FCGR3A genotype; *P* value represents the association between breast milk viral load and genotype determined by linear regression (D).

We next examined time to infant infection using Kaplan–Meier analyses to determine whether maternal FCGR3A genotype was associated with a particular transmission mechanism. In a log-rank test, there was a trend in association between maternal FCGR3A genotype and time to infant infection (*P* = .053). The Kaplan-Meier curves suggest that the majority of excess transmission risk in heterozygotes occurs during the peripartum period (Figure 1A). During this time, infections may be due to delivery or early breastfeeding. To address these possibilities, we dichotomized the mothers into breastfeeding (N = 188) and formula feeding (N = 191). The excess peripartum transmission risk in heterozygotes was observed in breastfeeding mothers (Figure 1B and C), but the effect was not statistically significant by log-rank analyses (*P* = .13). However, in a Cox proportional hazards model, a trend in heterozygotes having increased risk of transmission compared with homozygotes was observed (HR, 1.64; *P* = .064).

Because FcγR genotypes influence antibody effector functions (eg, ADCC) that may modulate viral load (reviewed in [1, 32, 33]), and because maternal breast milk viral load is a major risk factor for transmission [25], we hypothesized that FCGR3A genotype impacts breast milk viral load. Maternal FCGR3A heterozygotes (2.73 log_10_ copies/mL) did have slightly higher median breast milk viral loads than V/V (2.61 log_10_ copies/mL) and F/F (2.54 log_10_ copies/mL) homozygotes; however, in a linear regression model, maternal FCGR3A genotype was not associated with breast milk viral load (*P* = .37) (Figure 1D).

### Infant FCGR2A and FCGR3A Genotypes and Human Immunodeficiency Virus Progression

**Figure 2.**
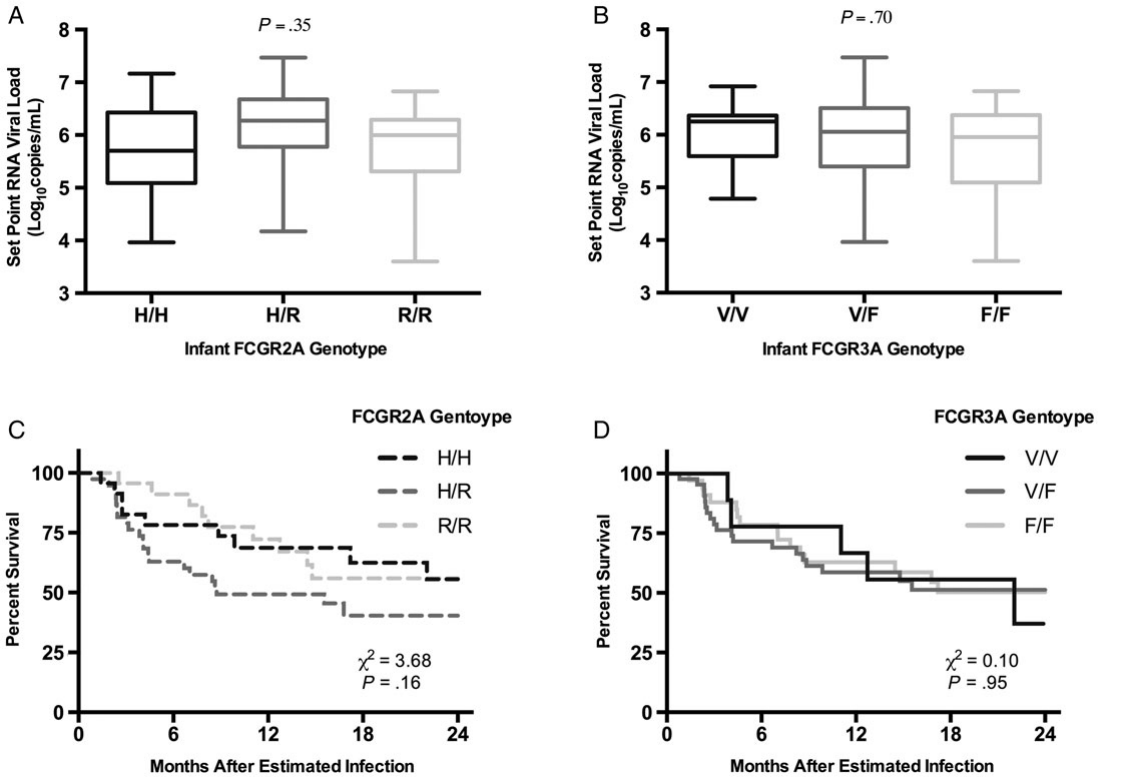
Infant FCGR2A and FCGR3A genotypes and disease progression in human immunodeficiency virus-infected infants. Infant set point viral load by infant FCGR2A (A) and FCGR3A (B) genotypes. *P* values represent the association between set point viral loads and genotype determined by linear regression. Kaplan–Meier estimates with log-rank statistics for survival after estimated infection by infant FCGR2A (C) and FCGR3A (D) genotypes.

Although infant FCGR2A and FCGR3A genotypes were not associated with infection risk, genotypes may alter disease progression in infected infants. Because passively acquired Fc-mediated ADCC activity has been associated with increased survival in HIV-infected infants in this cohort [22], we hypothesized that high-affinity FcγRs would be associated with slower disease progression. We examined both set point viral load and time to mortality after estimated infection as measures of disease progression. Set point viral loads were available for 49 (56.3%) infected infants [34]. In these infants, set point viral load was not associated with either FCGR2A (*P* = .35) or FCGR3A (*P* = .70) genotypes in a linear regression model (Figure 2A and B). FCGR2A (*P* = .16) and FCGR3A (*P* = .95) infant genotypes were also not associated with mortality after estimated infection (Figure 2C and D).

## DISCUSSION

In this study, we examined FCGR2A and FCGR3A genotypes in a cohort in which Fc-mediated responses are predictive of MTCT and disease progression [21, 22], thus allowing us to assess the relevance of FcγR genotype. Overall, infant genotypes were not associated with infant infection or progression, indicating that these polymorphisms may not be important predictors of HIV outcome. In mothers, FCGR2A genotype was not associated with transmission, but FCGR3A heterozygotes had an increased risk of MTCT.

Our finding that infant FCGR2A genotype does not impact infant infection risk differs from the one previous study of FCGR2A genotypes in MTCT that observed more infections in infants with the high-affinity receptor [14]. This prior study by Brouwer et al [14] was of similar sample size to our study, but it only considered perinatal infections. Similarly restricting our analyses, we did not observe a significant correlation between infant FCGR2A genotype and perinatal infection (data not shown). Therefore, the difference in results may be due to cohort differences, which included a restriction to women who were asymptomatic for HIV infection in the prior study. Thus, further studies are needed to clarify the impact of FCGR2A genotype on MTCT in different settings.

Infant genotype was also not associated with infected infant disease progression (set point viral load and mortality). These results support Brouwer et al [14] who similarly observed no association between FCGR2A and infant mortality. Overall, these data suggest that FCGR2A and FCGR3A genotypes likely do not substantially contribute to disease progression in HIV-infected infants.

The observation that infant FCGR2A and FCGR3A genotypes do not influence infant infection or disease progression has important implications for treatment and therapy. Given that Fc-mediated ADCC activity has been suggested to provide protective and/or therapeutic benefits (reviewed in [1]), our results suggest that host FcγR genotype may not impact vaccination or therapeutic regimens that depend on Fc-mediated antibody activities. Nonetheless, our study does not exclude the possibility that other FcγR SNPs, copy number variants, Fc genotypes (IgG allotypes), or posttranscriptional variation of these receptors may influence MTCT. For example, results from the RV144 vaccine trial, which showed evidence of protection in vaccinated adults, suggest that a FCGR2C polymorphism was associated with protection [20]. Although previous work in our laboratory observed a role of ADCC in infant outcome in a subset of the population studied here [21, 22], we were unable to analyze the collective effect of ADCC and FcγR genotypes because the ADCC studies were not performed with effector cells bearing individually matched FcγR genotypes of the mothers and infants. Future studies that examine ADCC using donor cells matched to host FcγR genotypes may help clarify the impact that these receptor genotypes have on ADCC activity and MTCT.

In mothers, FCGR2A genotype was not associated with transmission, but FCGR3A was associated with transmission risk. We unexpectedly found that FCGR3A heterozygotes (V/F) had increased risk of transmission compared with homozygotes (V/V or F/F). In particular, there was increased peripartum transmission in breastfeeding mothers, indicating that transmissions during the early breastfeeding period may be impacted by FCGR3A genotype. The biological mechanism for this heterozygote disadvantage is unclear. In vitro data vary, but they do not suggest that FCGR3A heterozygotes have lower antibody binding or functional (eg, ADCC) activity compared with the 2 homozygote groups [2, 4, 6, 35]. In addition, in our analyses, maternal FCGR3A genotype was not associated with breast milk viral load, a major risk factor for breastfeeding transmission [23, 25]. One older study by Lehrnbecher et al [19] observed higher rates of Kaposi's sarcoma (a marker for HIV progression) in FCGR3A heterozygotes compared with low-affinity homozygotes. These results suggest that maternal heterozygous may have more advanced disease and be more likely to transmit the virus; however, this hypothesis was not supported by maternal viral load data (another marker of progression), which showed similar viral loads across maternal genotypes. Because the significance of the heterozygote disadvantage observed in our study was modest, it is important that the results be verified in a larger cohort before efforts are made to define the mechanism.

## CONCLUSIONS

This study was the first to examine the impact of both FCGR2A and FCGR3A genotypes in HIV-infected mothers and their infants. Importantly, these receptor genotypes were studied in a population in which Fc-mediated ADCC activity has been shown to impact infant outcome [21, 22]. Overall, these results suggest that infant FcγR genotypes do not impact infection or disease progression. In mothers, there was some evidence that FCGR3A genotypes may impact transmission risk in the early breastfeeding period; however, more work will be needed to confirm this association and to explore potential mechanisms.

## References

[1] Lewis GK Role of Fc-mediated antibody function in protective immunity against HIV-1. Immunology 2014; 142:46–57. 24843871 10.1111/imm.12232 PMC3992047

[2] Forthal DN, Gilbert PB, Landucci G, Phan T Recombinant gp120 vaccine-induced antibodies inhibit clinical strains of HIV-1 in the presence of Fc receptor-bearing effector cells and correlate inversely with HIV infection rate. J Immunol 2007; 178:6596–603. 17475891 10.4049/jimmunol.178.10.6596

[3] Forthal DN, Landucci G, Bream J et al FcgammaRIIa genotype predicts progression of HIV infection. J Immunol 2007; 179:7916–23. 18025239 10.4049/jimmunol.179.11.7916

[4] Musolino A, Naldi N, Bortesi B et al Immunoglobulin G fragment C receptor polymorphisms and clinical efficacy of trastuzumab-based therapy in patients with HER-2/neu-positive metastatic breast cancer. J Clin Oncol 2008; 26:1789–96. 18347005 10.1200/JCO.2007.14.8957

[5] Bruhns P, Iannascoli B, England P et al Specificity and affinity of human Fcgamma receptors and their polymorphic variants for human IgG subclasses. Blood 2009; 113:3716–25. 19018092 10.1182/blood-2008-09-179754

[6] Dall'Ozzo S, Tartas S, Paintaud G et al Rituximab-dependent cytotoxicity by natural killer cells: influence of FCGR3A polymorphism on the concentration-effect relationship. Cancer Res 2004; 64:4664–9. 15231679 10.1158/0008-5472.CAN-03-2862

[7] Parren PW, Warmerdam PA, Boeije LC et al On the interaction of IgG subclasses with the low affinity Fc gamma RIIa (CD32) on human monocytes, neutrophils, and platelets. Analysis of a functional polymorphism to human IgG2. J Clin Invest 1992; 90:1537–46. 1401085 10.1172/JCI116022 PMC443201

[8] Clark MR, Clarkson SB, Ory PA et al Molecular basis for a polymorphism involving Fc receptor II on human monocytes. J Immunol 1989; 143:1731–4. 2527271

[9] Warmerdam PA, van de Winkel JG et al A single amino acid in the second Ig-like domain of the human Fc gamma receptor II is critical for human IgG2 binding. J Immunol 1991; 147:1338–43. 1831223

[10] Wu J, Edberg JC, Redecha PB et al A novel polymorphism of FcgammaRIIIa (CD16) alters receptor function and predisposes to autoimmune disease. J Clin Invest 1997; 100:1059–70. 9276722 10.1172/JCI119616 PMC508280

[11] Ravetch JV, Perussia B Alternative membrane forms of FcgammaRIIIa (CD16) on human natural killer cells and neutrophils. Cell type-specific expression of two genes that differ in single nucleotide substitutions. J Exp Med 1989; 170:481–97. 2526846 10.1084/jem.170.2.481 PMC2189395

[12] Poonia B, Kijak GH, Pauza CD High affinity allele for the gene of FCGR3A is risk factor for HIV infection and progression. PLoS One 2010; 5:e15562. 21187939 10.1371/journal.pone.0015562 PMC3004964

[13] Deepe RN, Kistner-Griffin E, Martin JN et al Epistatic interactions between Fc (GM) and FcgammaR genes and the host control of human immunodeficiency virus replication. Hum Immunol 2012; 73:263–6. 22213007 10.1016/j.humimm.2011.12.008 PMC3288776

[14] Brouwer KC, Lal RB, Mirel LB et al Polymorphism of Fc receptor IIa for IgG in infants is associated with susceptibility to perinatal HIV-1 infection. AIDS 2004; 18:1187–94. 15166534 10.1097/00002030-200405210-00012

[15] Forthal DN, Gabriel EE, Wang A et al Association of Fcgamma receptor IIIa genotype with the rate of HIV infection after gp120 vaccination. Blood 2012; 120:2836–42. 22915639 10.1182/blood-2012-05-431361 PMC3466964

[16] French MA, Tanaskovic S, Law MG et al Vaccine-induced IgG2 anti-HIV p24 is associated with control of HIV in patients with a ‘high-affinity’ FcgammaRIIa genotype. AIDS 2010; 24:1983–90. 20634666 10.1097/QAD.0b013e32833c1ce0

[17] Pandey JP, Namboodiri AM, Bu S et al Immunoglobulin genes and the acquisition of HIV infection in a randomized trial of recombinant adenovirus HIV vaccine. Virology 2013; 441:70–4. 23582638 10.1016/j.virol.2013.03.007 PMC3750738

[18] Weis JF, McClelland RS, Jaoko W et al Fc gamma receptors IIa and IIIa genetic polymorphisms do not predict HIV-1 disease progression in Kenyan women. AIDS Res Hum Retroviruses 2015; 31:288–92. 25312792 10.1089/aid.2014.0209 PMC4348085

[19] Lehrnbecher TL, Foster CB, Zhu S et al Variant genotypes of FcgammaRIIIA influence the development of Kaposi's sarcoma in HIV-infected men. Blood 2000; 95:2386–90. 10733511

[20] Li SS, Gilbert PB, Tomaras GD et al FCGR2C polymorphisms associate with HIV-1 vaccine protection in RV144 trial. J Clin Invest 2014; 124:3879–90. 25105367 10.1172/JCI75539 PMC4151214

[21] Mabuka J, Nduati R, Odem-Davis K et al HIV-specific antibodies capable of ADCC are common in breastmilk and are associated with reduced risk of transmission in women with high viral loads. PLoS Pathog 2012; 8:e1002739. 22719248 10.1371/journal.ppat.1002739 PMC3375288

[22] Milligan C, Richardson BA, John-Stewart G et al Passively acquired antibody-dependent cellular cytotoxicity (ADCC) activity in HIV-infected infants is associated with reduced mortality. Cell Host Microbe 2015; 17:500–6. 25856755 10.1016/j.chom.2015.03.002 PMC4392343

[23] Nduati R, John G, Mbori-Ngacha D et al Effect of breastfeeding and formula feeding on transmission of HIV-1: a randomized clinical trial. JAMA 2000; 283:1167–74. 10703779 10.1001/jama.283.9.1167

[24] John GC, Nduati RW, Mbori-Ngacha DA et al Correlates of mother-to-child human immunodeficiency virus type 1 (HIV-1) transmission: association with maternal plasma HIV-1 RNA load, genital HIV-1 DNA shedding, and breast infections. J Infect Dis 2001; 183:206–12. 11120927 10.1086/317918

[25] Rousseau CM, Nduati RW, Richardson BA et al Longitudinal analysis of human immunodeficiency virus type 1 RNA in breast milk and of its relationship to infant infection and maternal disease. J Infect Dis 2003; 187:741–7. 12599047 10.1086/374273 PMC3384731

[26] Benki S, McClelland RS, Emery S et al Quantification of genital human immunodeficiency virus type 1 (HIV-1) DNA in specimens from women with low plasma HIV-1 RNA levels typical of HIV-1 nontransmitters. J Clin Microbiol 2006; 44:4357–62. 17050820 10.1128/JCM.01481-06 PMC1698424

[27] John GC, Nduati RW, Mbori-Ngacha D et al Genital shedding of human immunodeficiency virus type 1 DNA during pregnancy: association with immunosuppression, abnormal cervical or vaginal discharge, and severe vitamin A deficiency. J Infect Dis 1997; 175:57–62. 8985196 10.1093/infdis/175.1.57 PMC3372419

[28] Nduati RW, John GC, Richardson BA et al Human immunodeficiency virus type 1-infected cells in breast milk: association with immunosuppression and vitamin A deficiency. J Infect Dis 1995; 172:1461–8. 7594703 10.1093/infdis/172.6.1461 PMC3358135

[29] Panteleeff DD, John G, Nduati R et al Rapid method for screening dried blood samples on filter paper for human immunodeficiency virus type 1 DNA. J Clin Microbiol 1999; 37:350–3. 9889216 10.1128/jcm.37.2.350-353.1999 PMC84304

[30] Savitz DA, Olshan AF Multiple comparisons and related issues in the interpretation of epidemiologic data. Am J Epidemiol 1995; 142:904–8. 7572970 10.1093/oxfordjournals.aje.a117737

[31] Van Der Pol WL, Jansen MD, Sluiter WJ et al Evidence for non-random distribution of Fcgamma receptor genotype combinations. Immunogenetics 2003; 55:240–6. 12830330 10.1007/s00251-003-0574-9

[32] Dijstelbloem HM, Van De Winkel JG, Kallenberg CG Inflammation in autoimmunity: receptors for IgG revisited. Trends Immunol 2001; 22:510–6. 11525942 10.1016/s1471-4906(01)02014-2

[33] Gillis C, Gouel-Cheron A, Jonsson F, Bruhns P Contribution of human FcγRs to disease with evidence from human polymorphisms and transgenic animal studies. Front Immunol 2014; 5:254. 24910634 10.3389/fimmu.2014.00254 PMC4038777

[34] Richardson BA, Mbori-Ngacha D, Lavreys L et al Comparison of human immunodeficiency virus type 1 viral loads in Kenyan women, men, and infants during primary and early infection. J Virol 2003; 77:7120–3. 12768032 10.1128/JVI.77.12.7120-7123.2003 PMC156211

[35] Congy-Jolivet N, Bolzec A, Ternant D et al Fc gamma RIIIa expression is not increased on natural killer cells expressing the Fc gamma RIIIa-158 V allotype. Cancer Res 2008; 68:976–80. 18281470 10.1158/0008-5472.CAN-07-6523

